# Advances in developing novel therapeutic strategies for Alzheimer’s disease

**DOI:** 10.1186/s13024-018-0299-8

**Published:** 2018-12-12

**Authors:** Jiqing Cao, Jianwei Hou, Jing Ping, Dongming Cai

**Affiliations:** 1 James J Peters VA Medical Center, Research & Development, Bronx, NY 10468 USA; 2 Department of Neurology, Alzheimer Disease Research Center, Icahn School of Medicine at Mount Sinai, New York, NY 10029 USA; 3 The Central Hospital of The Hua Zhong University of Science and Technology, Wuhan, China

**Keywords:** Alzheimer’s disease, Novel therapies, Pre-clinical and clinical trials, Drug development

## Abstract

Alzheimer’s Disease (AD), the most prevalent neurodegenerative disease of aging, affects one in eight older Americans. Nearly all drug treatments tested for AD today have failed to show any efficacy. There is a great need for therapies to prevent and/or slow the progression of AD. The major challenge in AD drug development is lack of clarity about the mechanisms underlying AD pathogenesis and pathophysiology. Several studies support the notion that AD is a multifactorial disease. While there is abundant evidence that amyloid plays a role in AD pathogenesis, other mechanisms have been implicated in AD such as tangle formation and spread, dysregulated protein degradation pathways, neuroinflammation, and loss of support by neurotrophic factors. Therefore, current paradigms of AD drug design have been shifted from single target approach (primarily amyloid-centric) to developing drugs targeted at multiple disease aspects, and from treating AD at later stages of disease progression to focusing on preventive strategies at early stages of disease development. Here, we summarize current strategies and new trends of AD drug development, including pre-clinical and clinical trials that target different aspects of disease (mechanism-based versus non-mechanism based, e.g. symptomatic treatments, lifestyle modifications and risk factor management).

## Background

Alzheimer’s disease (AD) is the most prevalent neurodegenerative disease of aging, and it affects over 26 million people worldwide with this number continuously increasing [1–3]. Nearly all drug treatments tested for AD today have failed to show any efficacy. There is a great need for therapies to prevent and/or slow the progression of AD.

AD is a complex disease with multifactorial etiology. Early-onset AD, a rare form of disease, follows an autosomal-dominant pattern in a majority of cases with mutations identified in amyloid precursor protein (APP), presenilin (PS) 1 and 2. Late-onset AD, a sporadic form of disease, affects over 90% of patients with several genetic loci and risk factors identified by a combination of genetic studies and bioinformatic approaches. These discoveries have shaped our current understanding of AD pathogenesis, as well as the development of therapeutic targets and design of clinical trials. While some of identified genetic risk factors can be linked to the most popular amyloid cascade hypothesis and the Tau theory [3], several commonly shared pathways have been implicated in AD such as immune system dysfunction, lipid and cholesterol dyshomeostasis, and vesicle trafficking and protein degradation pathway dysregulation [4–6]. Other theories have also been suggested such as mitochondrial dysfunction, lack of intrinsic support of neurotrophic factors, pre-existing comorbid medical conditions including cerebrovascular diseases, diabetes and hypertension, as well as environmental exposure such as virus infection [7–10].

Here we focus on current AD therapeutic strategies which comprise of mechanism-based approaches including amyloid-beta (Aβ) clearance, tau protein deposits, apolipoprotein-E (ApoE) function, neuroprotection and neuroinflammation, as well as non-mechanism based approaches including symptomatic cognitive stimulation, AD prevention, lifestyle modifications and risk factor management including non-pharmacological interventions (Table 1: a summary list of therapeutic strategies discussed in this paper).

**Table 1:** A summary of therapeutic approaches discussed in the paper

AD therapeutic strategies	
Mechanism-based Approaches	
1. Therapies Targeted at Amyloid 1.1 Inhibiting Aβ Production 1.2 Accelerating Aβ Clearance 1.3 Preventing Aβ Aggregation 2. Therapies Targeted at Tau 2.1 Tau Stabilizers and Aggregation Inhibitors 2.2 Therapies Targeted at Tau Post-translational Modifications 2.3 Anti-tau Immunotherapy 3. Therapies Targeted at ApoE 4. Neuroprotective Therapies 4.1 Neurotrophins and Their Receptor-based Therapies 4.2 Therapies Targeted at Neuroinflammation and Oxidative Stress	
Non-mechanism Based Approaches	
1. Symptomatic Cognitive Enhancers 2. Therapies and Interventions for AD Prevention 2.1 Secondary Prevention Interventions 2.2 Primary Prevention Interventions	

## Mechanism-based approaches

### Therapies targeted at amyloid

According to the amyloid cascade hypothesis, AD-related pathology typically begins with asymptomatic cerebral amyloidosis many years before the onset of clinical symptoms [11]. Accumulation of Aβ in the brain starts with monomeric Aβ leaving its reservoir in the cerebrospinal fluid (CSF) to form toxic aggregates followed by deposition on neuronal surface and synaptic terminals. Therefore, the majority of AD treatment strategies targeted at the amyloid cascade in the past 30 years or so has been focused on reducing Aβ generation through development of β- and γ-secretase inhibitors, accelerating Aβ clearance through active and passive immunotherapies, as well as preventing formation of toxic amyloid aggregates.

#### Reducing Aβ generation

Amyloid Aβ is derived from APP cleaved by two membrane-bound enzymes, β-secretase and γ-secretase complex. Therefore, modulation of these enzymes to inhibit Aβ production has been a major focus in developing AD therapies. The development of β-site APP cleaving enzyme 1 (BACE1) inhibitors was limited at the beginning due to difficulties in drug delivery. Later brain-penetrant BACE1 inhibitors have been developed with data showing promising efficacy at reducing Aβ in animal models [12, 13]. However, most BACE1 inhibitors tested today failed to survive beyond phase II/III clinical trials due to either lack of efficacy, or undesirable long-term side effects (Table 2: a summary list of AD drugs tested in clinical trials). For example, Merck halted its ongoing clinical trials of verubecestat (MK-8931) in mild to moderate AD patients [14–16], and most recently in people with prodromal AD (the APECS: β amyloid Production and Effects on Cognition Study; NCT01953601). However, despite the disappointing results from current clinical trials of BACE1 inhibitors, a recent study demonstrated that conditional knockout of BACE1 was capable of completely reversing pre-formed amyloid deposition and improving cognitive function in a mouse model with 5× Familial AD (FAD) transgenic background, suggesting sequential and gradual inhibition of BACE1 could be beneficial for AD patients [17]. It was pointed out that BACE1 is necessary to maintain optimal cognitive function and that the BACE1 inhibition is not without concerns [17]. More studies are needed to clarify the mechanism(s) of BACE inhibitors in AD, to determine optimal timing for BACE1 inhibition in adult AD patients, and to search for drug candidates without unwanted and off-target toxicities.

**Table 2:** A summary list of AD drugs and therapies tested in clinical trials

AD drugs tested in clinical trials					
Drug	Phase	Subject	NCT	Summary	Reference
1.Therapies Targeted at Amyloid					
1.1 Reducing Aβ Generation					
MK-8931 (BACE inh.)	III	Prodromal AD	NCT01953601	lack of efficacy	14-16
LY450139	III	Mild to moderate AD	NCT00762411; NCT00594568	lack of efficacy	15,18, 19,
(γ-secretase inh.)			NCT01035138		34, 36, 37
Avagacestat	II	Prodromal AD	NCT00890890	no efficacy	35
NIC5-15	II	Probable AD	NCT01928420	completed	15,40
R-flurbiprofen	III	Probable AD	NCT00105547; NCT00322036	lack of efficacy	42
EVP-0962	II	Healthy, MCI or early AD	NCT01661673	terminated	15
1.2 Accelerating Aβ Clearance					
AN-1792	II	Mild-to-moderate AD	NCT00021723	severe meningoencephalitis	44
Affitope AD02	II	Early AD	NCT02008513; NCT01117818	no efficacy	46
CAD106	II/III	Mild AD	NCT02565511	ongoing	15, 47
Bapineuzumab	III	Mild-to-moderate AD	NCT00667810; NCT00575055 NCT00574132	no efficacy	43, 48
Solanezumab	III	Mild-to-moderate AD probable AD	NCT00905372; NCT00904683 NCT01900665	no efficacy	43, 49
BAN2401	II	MCI due to AD and mild AD	NCT01767311	positive results	50
Crenezumab	III	Probable AD or prodromal AD	NCT03114657; NCT02670083	ongoing	
Gantenerumab	III	Probable AD or prodromal AD	NCT03443973; NCT03444870	ongoing	
Aducanumab	I	Prodromal or Mild AD	NCT01677572	positive results but ARIA	51
	III	MCI due to AD or mild AD	NCT02484547; NCT02477800	ongoing	
1.3 Other Anti-amyloidogenic Compounds with Diverse Mechanisms of Action					
ALZT-OP1	III	Early AD	NCT02547818	ongoing	43
GV-971	III	Mild-to-moderate AD	NCT02293915	completed	43
Posiphen	I	MCI or probably AD	NCT02925650	ongoing	52
ELND005	II/III	Mild-to-severe AD		ongoing	53
ALZ801	III	Mild AD (ApoE4 carriers)		ongoing	43
2. Therapies Targeted at Tau					
2.1 Tau Stabilizers and Aggregation Inhibitors					
TPI 287	I	Probable AD	NCT01966666	ongoing	
Rember™	II	Mild or moderate AD	NCT00684944; NCT00515333	no efficacy	62, 63
TRx0237	III	Mild-to-moderate AD/BvFTD	NCT01689233; NCT01689246 NCT02245568	no efficacy	64
TauRx	II/III	Mild or moderate AD	NCT03539380	ongoing	
2.2 Therapies Targeted at Tau Post-translational Modifications					
Lithium and Valproate	II	AD	NCT00088387	no efficacy	67, 68
NP-12	IIb	Mild-to-moderate AD	NCT01350362	no efficacy	69-71
2.3 Anti-tau Immunotherapy					
AADvac1	II	Mild-to-moderate AD	NCT02579252	ongoing	94
ACI-35	I	Mild-to-moderate AD	ISRCTN13033912	completed	61
ABBV-8E12	II	PSP; MCI or probable AD	NCT03391765; NCT02880956	ongoing	
RO7105705	I	Healthy	NCT02820896	ongoing	
3. Therapies Targeted at ApoE					
Bexarotene	II	Probable AD	NCT01782742	no efficacy	142
4. Neuroprotective Therapies					
4.1 Neurotrophins and Their Receptor-based Therapies					
NGF	I	Probable early AD	NCT00017940	positive results	170
AAV2-NGF	II	Mild-to-moderate AD	NCT00876863	no efficacy	173
LM11A-31 (p75 inh.)	I/II	Mild-to-moderate AD	NCT03069014	ongoing	
4.2 Therapies Targeted at Neuroinflammation and Oxidative Stress					
Dimebon	III	AD	NCT00912288	no efficacy	209
valacyclovir	II	Probable AD	NCT03282916	ongoing	
5. Symptomatic Cognitive Enhancers					
Idalopirdine	II	Probable AD	NCT01019421	positive results	233
(5-HT6 antag.)	III	Mild-to-moderate AD	NCT01955161; NCT02006641 NCT02006654	no efficacy	234
GSK239512 (H3R antag.)	II	Probable AD	NCT01009255	no efficacy	235
ABT288 (H3R antag.)	II	Mild-to-moderate AD	NCT01018875	no efficacy	236
Rasagiline (MAOB inh.)	II	Probable AD	NCT02359552	ongoing	237
Ladostigil (combined)	II	MCI or Mild-to-moderate AD	NCT01429623; NCT01354691	no efficacy	238-241
AZD0530	Ib	Mild-to-moderate AD	NCT01864655	safety and tolerance	252
(Fyn kinase inh.)	II	Mild AD	NCT02167256	ongoing	
Cilostazol	II	MCI	NCT02491268	positive results	255-257
(PDE3 inh.)	IV	Mild-to-moderate AD	NCT01409564	ongoing	
HT-0712 (PDE4 inh.)	II	Age-associated memory impairment	NCT02013310	completed	253, 254
Roflumilast (PDE4 inh.)	I	Scopolamine-induced CI	NCT02051335	no efficacy	253, 254
	II	Healthy	NCT01433666	positive results	253, 254
		Age-associated memory impairment	ISRCTN96013814	completed	253, 254
BPN14770 (PDE4 inh.)	I	Healthy	NCT02648672; NCT02840279	positive results	253, 254
BI 409306 (PDE9 inh.)	I	Healthy	NCT01343706	safety and tolerance	253
	II	MCI due to AD and mild AD	NCT02337907	ongoing	253, 254
PF044467943	I	Mild-to-moderate AD	NCT00988598	safety and tolerance	253
(PDE9 inh.)	II	Mild-to-moderate AD		no efficacy	253
6. Therapies and Interventions for AD Prevention					
PROSPER (statin)	II	High risk (with AD parents)	NCT00939822	no efficacy	265
ACCORD-MIND	III	DM2	NCT00182910	no efficacy	268
SNIFF (i.n. insulin)	II; II/III	MCI or AD or probable AD	NCT00438568; NCT01767909	ongoing	271-273
Metformin	IV	Age >60 years with IGT	NCT02432287	completed	274
Pioglitazone	II	Mild-to-moderate AD	NCT00982202	safety and tolerance	275
MIND (diet)		BMI≥25 non-demented	NCT02817074	ongoing	277, 278
FABS (fitness)		Non-demented	ACTRN12605000136606	positive results	280
ACTIVE (cog. training)	II/III	MCI	NCT00298558	positive results	282
Vit. E + Memantine	III	Mild to moderate AD	NCT00235716	positive results	284, 285
Ginkgo biloba	III	Non-demented and MCI	NCT00010803	no efficacy	286, 287
EGb 761®	IV	Subjects with memory complaints	NCT00276510	no efficacy	288
MIDAS		Age-associated memory impairment	NCT00278135	positive results	290
FINGER		High risk	NCT01041989	positive results	291
MIND-ADMINI		Prodromal AD	NCT03249688	ongoing	

The γ-secretase complex is comprised of four subunits [18, 19], with presenilin (PS) exhibiting catalytic activities of γ -secretase [20–28]. There is a long list of γ-secretase substrates with APP and Notch among the most well-known due to their implications in AD and cancer [18, 29]. Substantial effort has been invested into developing small-molecule inhibitors of γ-secretase for AD therapies. Non-selective γ-secretase inhibitors led to a decrease in brain Aβ [30, 31], and reduced Notch signaling at the same time causing gastrointestinal (GI) symptoms and compromised immune system [32, 33]. Despite the concerns, non-selective γ-secretase inhibitors such as semagacestat were tested in clinical trials (see in Table 2; NCT00762411, NCT00594568, NCT01035138), but discontinued at Phase III stage because of lack of efficacy or even worsened cognitive performance, and patient intolerance due to severe off-target side effects like GI irritation and skin cancer [15, 18, 19, 34–37]. Selective γ-secretase inhibitors include Notch-sparing γ-secretase inhibitors and γ-secretase modulators. Gleevec, the abl kinase inhibitor was found to reduce Aβ production but spare Notch cleavage by γ-secretase in primary neuronal cells and animals [38]. Avagacestat was reported to inhibit APP processing more preferably than Notch cleavage [39]. However, Phase II clinical trial of Avagacestat was discontinued due to adverse side effects suggesting possible Notch inhibition just like Semagacestat ([35]; NCT00890890). A recently developed Notch-sparing γ-secretase inhibitor pinitol (NIC5–15) was derived from natural product and reported to have insulin sensitization property. It is currently in Phase II trial for the treatment of AD ([15, 40]; NCT01928420). Non-steroidal anti-inflammatory drugs (NSAIDs) were the first γ-secretase modulators shown to shift Aβ production from the aggregable form (Aβ_42_) to a more soluble form (Aβ_38_) [41]. One of these NSAIDs, R-flurbiprofen [42] failed to exhibit any efficacy in Phase III trial of mild AD subjects (NCT00105547, NCT00322036). A γ-secretase modulator EVP-0962 showed potency in preclinical stage but no efficacy in Phase II trial of mild cognitive impairment (MCI) or early AD subjects [15] (NCT01661673).

#### Accelerating Aβ clearance

On the other hand, immunotherapy via active and passive vaccines against Aβ has been utilized as a therapeutic approach in AD [43]. The clinical trials of active immunotherapy targeted at Aβ failed until now due to autoimmune responses. The first active immunization clinical study in mild to moderate AD patients was discontinued in Phase II because a small portion of cohort developed severe meningoencephalitis with the AN-1792 vaccine targeting full-length Aβ_1–42_ peptide (NCT00021723) [44]. Subsequent development of vaccines was tailored to target specific Aβ epitopes such as ACC-001 which targets an N-terminal Aβ_1–7_ peptide fragment [15, 45]. Currently 2 s-generation active immunotherapy vaccines have been progressed into clinical trials (Affitope AD02 terminated in Phase II study [46], NCT02008513; CAD106 [15, 47] still in Phase II/III study, NCT02565511).

Comparing to active vaccination, passive anti-Aβ immunization may be a more promising strategy for treating AD. Unfortunately, most currently available data in clinical trials fail to meet the primary endpoints. Bapineuzumab is an antibody targeted at the N-terminus of Aβ peptide acting on soluble Aβ. However, no beneficial effect was found with Bapineuzumab in Phase III clinical studies of mild-to-moderate AD subjects [43, 48] (NCT00667810; NCT00575055; NCT00574132). Similarly, Solanezumab targeted at Aβ_16–24_ epitope, which was reported to only recognize soluble instead of fibrillary Aβ, failed in Phase III trial of mild AD patients [43, 49] (NCT00905372; NCT00904683; NCT01900665). BAN2401 targeted at soluble Aβ protofibrils, recently completed a Phase II trial of 856 MCI or mild AD subjects with confirmed brain amyloid pathology [50] (NCT01767311) with positive results from the highest dosage treatment group (10 mg/kg bi-weekly). It was reported that the statistical significance was achieved on key efficacy endpoints after 18 months of high dose treatment on slowing clinical progression measured by the Alzheimer’s Disease Composite Score (ADCOMS) and the Alzheimer’s Disease Assessment Scale-Cognitive Subscale (ADAS-Cog) score, as well as on reducing brain amyloid accumulation determined by amyloid-PET. However, those who interpret these results must be cautious as most of the ApoE4^+^ carriers were removed from the high-dose treatment group due to concerns of developing amyloid-related imaging abnormalities-edema (ARIA-E; based on AAIC 2018 Conference news). The full data set including the detailed demographic information of treatment versus placebo groups has yet to be shared with the scientific community.

Crenezumab (NCT03114657; NCT02670083), Gantenerumab (NCT03443973; NCT03444870), and Aducanumab (NCT02484547; NCT02477800) targeted both soluble and aggregated Aβ species (both oligomeric and fibrillar Aβ), are currently in Phase III trials in patients with prodromal, mild, and early AD, respectively. The results from preclinical and Phase 1b clinical trial studies regarding Aducanumab demonstrated that treatment reduced amyloid plaque levels in prodromal and mild AD patients in a dose-dependent manner. Additionally, cognitive results from clinical dementia rating-sum of the boxes scale (CDR-SB) and mini-mental status examination (MMSE) taken at the 54th week of treatment demonstrated that Aducanumab confers delayed cognitive decline. The main safety and tolerability concerns were ARIA [51] (NCT01677572).

#### Other anti-amyloidogenic compounds with diverse mechanisms of action

In addition to enzyme inhibitors and vaccines, there are other compounds developed with diverse mechanisms of action. ALZT-OP1, a combination therapy of two drugs with efficacy at preventing Aβ aggregation and neuroinflammation, is currently in Phase III clinical trial [43] (NCT02547818). GV-971, an oral sodium oligo-mannurarate with the ability to reduce Aβ toxicity in vitro, is in Phase III clinical trial of mild-to-moderate AD patients [43] (NCT02293915). Several low-molecular-weight anti-amyloidogenic drugs are currently in clinical investigations, e.g. Posiphen in Phase I/II trial for the treatment of MCI, AD and Parkinson’s disease (PD) [52] (NCT02925650), ELND005 in Phase II/III trial for treatment of AD type dementia [53] (NCT00934050), and ALZ-801 in Phase III clinical trial for AD [43].

The current belief in the field about the failure of anti-Aβ therapies indicates that late stage of interventions failed to provide full effects. Ongoing clinical trials of passive immunotherapy are focusing on targeting prodromal AD study cohorts with the goal to test anti-amyloid treatments in “correct” populations. This will be discussed in detail in the following section of “Therapies and Interventions for AD Prevention”. On the other hand, active Aβ immunotherapy as an effective way to prevent AD remains to be tested, and many safety concerns such as the severity of immune responses against the vaccine need to be investigated further.

### Therapies targeted at tau

Tau is a microtubule-binding protein which forms neurofibrillary tangles (NFTs), another neuropathological hallmark of AD [54, 55]. Interestingly, previous reports suggest that tau deposit correlates better with cognitive decline than amyloid plaque does [56], and that Aβ-induced neuro-toxicity is mediated by tau hyper-phosphorylation through a toxic gain-of-function effect [57–60]. Because of many recent failures in anti-Aβ drug trials, therapies targeted at tau have come into focus in AD drug development field. Current therapeutic strategies are categorized into inhibiting tau aggregation, reducing hyper-phosphorylation or other toxic post-translational modifications of tau, as well as promoting tau clearance and preventing tau spread [61].

#### Tau stabilizers and aggregation inhibitors

Most tau stabilizing agents manifested undesirable toxic side effects, e.g. paclitaxel and epothilone D [15]. A recent Phase I clinical trial study of a tau stabilizer TPI 287 in mild-to-moderate AD, progressive supranuclear palsy (PSP), and corticobasal syndrome (CBS) patients, conveyed encouraging results with beneficial effects of TP1 287 on cognitive performance and/or nerve cells activity (based on CTAD 2017 conference news). The trial is still ongoing with more safety and exploratory clinical effects to be analyzed (NCT01966666).

In parallel, several tau aggregation inhibitors tested have failed in clinical trials due to either unwanted side effects or lack of efficacy, e.g. Rember™ in Phase II [62, 63] (NCT00684944; NCT00515333), and TRx0237 (LMTM) in three Phase III trials of patients with mild-to-moderate AD and behavioral variant frontotemporal dementia [64] (NCT01689246; NCT01689233; NCT02245568). An ongoing Phase II/III trial by TauRx, aiming to enroll people with all-cause dementia and AD from multiple sites, will compare a 6-month course of 4 mg of LMTM twice daily to a different kind of placebo. Primary outcomes include ^18^F- fludeoxyglucose positron emission tomography (FDG-PET) imaging and safety; secondary outcomes include structural magnetic resonance imaging (MRI), as well as measures of cognition and activities of daily living (ADL; NCT03539380).

#### Therapies targeted at tau post-translational modifications

Another aspect of tau targeted therapies focuses on toxic post-translational modifications of tau: 1) inhibiting tau hyper-phosphorylation kinases such as glycogen synthase kinase 3 beta (GSK3훽) and cyclin dependent kinase 5 (CDK5); 2) promoting the activity of tau dephosphorylation enzyme protein phosphate 2A (PP2A) [65]; 3) modulating tau acetylation and cis-transformation [66].

There have been no success in clinical trial studies of GSK3훽 inhibitors, e.g. lithium and valproate in Phase II trial of AD patients [67, 68] (NCT00088387), and NP-031112 (NP-12) in Phase IIb trial [69–71] (NCT01350362). Development of other GSK3훽 inhibitors like paullone has not progressed past the preclinical trials due to concerns with cytotoxic effects [72, 73]. Unfortunately, there are no tau kinase inhibitors other than GSK3 inhibitors that have entered clinical trials due to several limiting factors such as kinase specificities and unclear safety profiles. Most selective inhibitors of CDK5 with preclinical efficacy [74, 75] have yet to be tested in clinical trials due to uncertainty of how selective these inhibitors are over other CDK family members as well as poor understanding of safety risks associated with CDK5 inhibition in humans. The results of other inhibitors of tau kinases like JNK and DYRKIA were disappointing with either negative clinical outcome measures or severe adverse side effects [66].

Besides tau hyperphosphorylation, other tau post-translational modifications such as acetylation have been implicated in AD and related tauopathies [76–78]. Therapeutic approaches such as limiting tau K280/K281 acetylation [79] or reducing tau acetylation by protein deacetylase SIRT1 [80] could restore microtubule stability and/or ameliorate tau-associated neurodegeneration in animal models. Whether targeting tau acetylation could be a feasible therapeutic approach for AD or other tauopathies is yet to be tested in clinical studies. Furthermore, studies by Lu and colleagues demonstrated that cis-p-tau transformation causes gain-of-toxic conformational changes of tau leading to aggregation, and that a monoclonal antibody against cis-p-tau can block this phenomenon [81–83]. Another strategy is to inhibit O-GlcNAcase, an enzyme that strips sugars from tau. It is believed that O-GlcNAcylation either competes with phosphorylation for the same serine/threonine residues, or simply prevents tau molecules from cozying up to one another. In animal studies, O-GlcNAcase inhibitors suppress tau phosphorylation, prevent tangles, and boost neuronal survival [84, 85]. Further studies are needed to determine the clinical efficacy of these strategies.

#### Anti-tau immunotherapy

The basis for anti-tau immunotherapy stems upon the discovery of trans-cellular tau spread with evidence supported by studies in mouse models as well as in clinical settings [86–89]. The rationale behind the application of immunization against pathologic tau variants is to interrupt the uptake and propagation processes of abnormal tau [90]. Using high-affinity antibody against phosphorylated tau is an approach that hopefully would not disturb the function of physical tau from active immunization of phosphorylated tau [91].

Several studies using active immunization against phospho-tau peptides modulating tau pathology showed positive results in tauopathy or AD mouse models [92, 93]. Currently, two active vaccines (AADvac1 and ACI-35) are being tested in clinical trials with AD patients [15]. AADvac-1 is a synthetic peptide derived from tau for active immunization which is going into Phase II trials [94] (NCT02579252). ACI-35 is a liposome-based 16-amino acid, tetra-palmitoylated phospho-tau peptide being tested in people with mild to moderate AD during Phase I study [61] (ISRCTN13033912). However, several concerns about active immunization of tau remain to be addressed such as the development of immune responses to other regions of tau epitope also known as epitope spread, and the likelihood of irreversible active immunization processes [64].

Tau-targeted passive immunization is an exciting development in AD because it offers the possibility of halting the spread of tau pathology through the extracellular space. In some tauopathy mouse models, certain tau antibodies halt the progression of NFT pathology even after onset of tangle formation [95–98]. Currently, ABBV-8E12, an antibody against aggregated, extracellular tau, is in two Phase II trials targeting PSP patients and MCI subjects with positive amyloid scan (NCT03391765; NCT02880956). RO7105705, a pan-tau antibody targeted at N-terminus of all six isoforms of human tau, is now in Phase I trial which evaluates once weekly 8400 mg doses of the antibody in healthy controls and AD patients (NCT02820896; AAIC 2017 Conference News). Several other tau antibody candidates are heading toward Phase I trials. However, concerns of autoimmunity against endogenous tau just like Aβ immunotherapy remain to be addressed.

The link between tau pathology and cognitive dysfunction raises the hope that cognitive decline can be slowed or even halted by therapies targeted at tau. Strategies discussed in this section include stabilizing microtubules, reducing tau aggregation, inhibiting hyperphosphorylation or other toxic modifications of tau, as well as active and passive immunization against tau to promote clearance and interrupt transcellular spread. Other approaches that upregulate the clearance and degradation of tau through the ubiquitin/proteasome system and the autophagy/lysosome pathway would likely have favorable effects as well, which are not discussed in this section.

### Therapies targeted at ApoE

The ApoE4 genotype is one of the strongest genetic risk factors for developing sporadic AD [99]. On the other hand, those with ApoE2 genotype demonstrate a lower risk of developing AD and a delayed age of onset regarding AD symptoms. Several Aβ-dependent [100–102] and Aβ-independent mechanisms [103, 104] have been proposed for ApoE in AD pathogenesis. Different therapeutic strategies targeted at ApoE were tested in vitro and/or in vivo preclinical animal models including blocking Aβ-ApoE interaction by small peptide fragments [105–108], manipulating ApoE levels (drug treatments to stimulate ApoE expression [109], or decrease ApoE expression [110]), viral delivery of ApoE2 [111, 112], ApoE antibodies [113, 114], structural modifiers [115–117], lipidation promoting compounds [118–121] and ApoE mimetic peptides [122–125].

Blocking the interaction between ApoE and Aβ by a peptide fragment of Aβ reduced brain amyloid accumulation, ameliorated memory deficits [105, 107], and reduced brain insoluble tau levels [126] in AD transgenic mouse models. Interestingly, ApoE immunotherapy can achieve similar effects on decreasing amyloid burden [113, 114]. One possible mechanism of action is blocking the ApoE-Aβ interaction by anti-ApoE antibodies. When given at pre-plaque stage [113] or after plaque deposition [114], an anti-mouse ApoE antibody decreased amyloid plaque load and improved brain functional connectivity and cognition in an APP transgenic mouse model. No overt adverse side effects such as changes in total cholesterol or cerebral amyloid angiopathy load were observed with anti-ApoE therapy in mice [113, 114]. It should be noted that severe dyslipidemia associated with ApoE deficiency [127] needs to be carefully examined before moving forward into clinical applications.

Regulation of ApoE quantity is one of the main therapeutic approaches tested for AD. However, conflicting results have been reported from clinical studies for AD biomarkers comparing ApoE levels of AD patients with normal individuals [128–131]. Interestingly in animal models, decreasing expression of ApoE3 or ApoE4, or increasing expression of ApoE2 lowered brain amyloid deposition [111, 112]. Numerous animal studies have also been performed to evaluate the therapeutic potential of compounds that increase brain ApoE levels [109, 118, 120, 121, 132–141]. For example, oral administration of bexarotene, an agonist of retinoid X receptors (RXRs) which positively regulates ApoE transcription, was found to increase brain ApoE, reduce Aβ deposition and improve cognitive function in an AD transgenic mouse model [109]. However, a later report failed to replicate the beneficial effects of bexarotene in mouse models [137]. When juxtaposed with the adverse side effects of bexarotene including hepatic failure and lack of efficacy in reducing brain amyloid load from a Phase II clinical trial of AD patients (NCT01782742), the enthusiasm for this drug diminished [142].

On the other hand, effects of viral delivery of human ApoE assessed in APP transgenic mice showed reduced amyloid plaque load by ApoE2 and increased plaque load by ApoE4 [111, 112]. Similar effects on mouse Aβ levels were observed in ApoE4 targeted replacement (TR) mice without human APP transgenic background [143]. Overall, results from animal studies suggest that gene therapy increasing ApoE2 expression might be beneficial [144]. Further studies are critical to determine gene therapy strategies for ApoE4 conditions, e.g. gene silencing versus increasing expression [144].

Another line of approach targeted at ApoE in AD is through the modification of ApoE properties such as ApoE structural modifiers and lipidation-promoting agents. It was reported that the interaction between residues Arg61 and Glu255 of ApoE4 confers abnormal structural conformation associated with ApoE4-induced neurotoxicity [145, 146]. Therefore, a potential therapeutic approach is to modify the pathological structure of ApoE4. For example, a recent report using human neurons derived from induced pluripotent stem cells of ApoE4 subjects, demonstrated that treatment with a small-molecule structure corrector of ApoE4 reduced levels of ApoE4 fragments, increased numbers of GABAergic neurons, as well as reduced production and/or secretion of p-tau, Aβ_40_ and Aβ_42_ [117].

Moreover, lipidation of ApoE significantly affects its function [147] and differential properties of lipidation between ApoE isoforms have been reported. ApoE4 is found to be poorly lipidated in humans [148] and in ApoE mice [149, 150], and promoting ApoE4 lipidation through activation of nuclear receptor pathways (the LXR/RXR-ABCA1 axis) could be a therapeutic strategy [140, 151, 152]. However, one potential concern is the likelihood of increasing ApoE4 levels and exacerbating the detrimental effects of ApoE4 [104]. Future work is needed to explore how ApoE lipidation and function are regulated by brain lipid homeostasis [153].

It should be noted that the ApoE4 genotype is an important determinant of therapeutic responses in AD clinical trials [154]. For example, Phase III trials of bapinuezumab showed significant differences in treatment responses between ApoE4^+^ and ApoE4^−^ AD patients [155]. Similarly, data from the Cardiovascular Health Cognition Study indicated that protective effects of nonsteroidal anti-inflammatory drugs (NSAIDs) against AD development, can only be seen in 65 years or older ApoE4^+^ individuals [156]. More importantly, the risk for developing AD was significantly higher in ApoE4^+^ women than in ApoE4^+^ men [157–159]. Collectively, the potential impact of ApoE4 genotype and its interaction with sex need to be carefully considered when designing clinical trials to evaluate therapeutic efficacy in AD.

### Neuroprotective therapies

In AD, synaptic dysfunction caused by a combination of factors including accumulation of toxic aggregates, age-related processes, and neuroinflammation is one of disease’s hallmarks. While the majority of AD therapeutic efforts advanced in clinical trials have been focused on targeting amyloid and tau, neuroprotective strategies are developed to target at degenerative mechanisms triggered by or involving factors mentioned above.

#### Neurotrophins and their receptor-based therapies

Neurotrophins (NTs) and their receptor-based therapies in AD have been explored for years because of the pleiotropic actions of NTs and receptor signaling [160–166]. However, suboptimal pharmacological profiles of NTs such as short plasma half-lives, poor oral bioavailability and BBB permeability, as well as limited brain tissue diffusion, limit their clinical application [167–169]. Alternatively, gene transfer technology has been utilized in developing NTs-based AD therapy.

A phase I clinical trial of implantation of genetically modified autologous fibroblasts expressing nerve growth factor (NGF) into the basal forebrain area of eight mild AD patients demonstrated efficacy at improving the cognitive decline rate and increasing brain metabolic activity measured by serial 18-fluorodeoxyglucose PET scans [170] (NCT00017940). Autopsied brains confirmed sustained expression and activity of viral-delivered NGF [171]. In addition, it was observed that axons sprouted toward the local source of NGF and cell hypertrophy [172]. Subsequent Phase II trial recruited 49 mild to moderate AD subjects who were randomly assigned to receive intracerebral injections of AAV2-NGF or sham surgery. While AAV2-NGF delivery was well-tolerated, it did not affect clinical outcomes, or selected AD biomarkers 2 years after NGF delivery [173] (NCT00876863). It was argued that these results were inconclusive due to a small sample size. In addition, whether viral delivery achieved the targeted expression of NGF in treatment group needs to be evaluated.

NT receptor-based therapies focus on targeting p75, Tropomyosin Receptor Kinase A (TrkA) and TrkB receptors. The goal of p75-targeted therapeutic strategies is to develop small molecules that can inhibit degenerative signaling of p75 [174, 175], and promote survival even in the absence of NTs [175, 176]. A brain-penetrant small molecule, called LMA11A-31, counteracts the toxicity of Aβ and tau to prevent synaptic dysfunction, spine loss, neurite degeneration, microglia activation, and cognitive deficits in animal models [177, 178]. This compound reversed degeneration in 12-month-old AD mice [179], and repaired age-related loss of cholinergic neurons in wild-type mice (CTAD 2017 meeting report). In a Phase I trial, single or multiple doses of LM11A-31 were well tolerated by young or old volunteers with no adverse events reported. An ongoing Phase IIa trial targeted at mild to moderate AD patients will evaluate the effects of LM11A-31 at two dosages (NCT03069014).

Previous conflicting reports implicate the complexity of targeting TrkA for AD therapies, e.g. results showing positive effects of TrkA activation in cellular and mouse models of AD [180, 181] versus data showing beneficial effects of TrkA inhibition [182, 183]. A series of small-molecule ligands have been developed that either bind to and activate TrkB alone, or both TrkB and TrkC as well as downstream effectors of the signaling pathways, demonstrating some beneficial effects in vitro and in wildtype animals [175, 184, 185]. Systemic injection of a TrkB agonist 7,8-dihydroxyflavone (7,8-DHF) reduced brain amyloid load, prevented synaptic loss, and rescued cognitive deficits in 5XFAD mice [186–188]. However, later work failed to show efficacy at reducing pathology or improving cognitive deficits in the APP/PS1 transgenic mouse model with concerns regarding limited bioavailability of 7,8-DHF [189]. Together, NTs and receptor-based therapies have some potential for synergistic intracellular signaling [190], and the feasibility to combat multifaceted pathological mechanisms of AD but these strategies needs to be carefully evaluated before clinical applications.

#### Therapies targeted at Neuroinflammation and oxidative stress

The role of neuroinflammation and involvement of microglia in AD pathogenesis have been increasingly recognized and supported by a large amount of evidence including genetic studies, data mining and multiscale network analysis [191–194]. The microglial priming model proposes that during pre-symptomatic stage of AD, microglia is activated by proinflammatory mediators [192], and subsequently astrocytes acquire a proinflammatory phenotype amplifying neuronal damage [195–199]. The glial dysfunction may be independent from the presence of Aβ and tau at early stages of disease, leading to synaptic dysfunction and neuronal death [200, 201]. Therefore, molecules that restore physiological function of microglia and astrocytes may offer new directions for AD therapy.

Different strategies are developed at modulating immune cell function in neuroinflammation such as reducing gene expression of cytokines, inhibiting cytokine release and preventing cytokines binding to their receptors [202]. Molecules such as minocycline with anti-inflammatory properties have been shown to reduce cytokine release from astrocytes, and thereby rescue cognitive deficits in AD mouse models [203, 204]. Interestingly, it was found that inhibiting tau phosphorylation kinases such as GSK3*β* could also achieve satisfactory results of modulating neuroinflammation in animal models [205]. Reduced oxidative injury is another neuroprotective approach. For example, inhibition of cyclooxygenase-2 and inducible nitric oxide synthase has positive outcomes based on in vitro and in vivo animal studies [206–208]. While mitochondrial enhancers like Dimebon (latrepirdine) failed to show efficacy in clinical trials [209] (NCT 00912288), effort continues at searching for more effective agents targeted mitochondrial dysfunction with the hope to restore synaptic and neuronal function [210, 211].

Another strategy targeted at microglial function is through experimental manipulations to promote microglial encapsulation of amyloid plaques and reduce axonal dystrophy [212]. Several groups have shown that passive immunization of anti-Aβ antibody [51, 212, 213], or anti-ApoE antibody [113] increases recruitment of microglia around amyloid plaques in AD mouse models. Genetic deletion of the chemokine receptor CX3CR1 in microglia can also enhance formation of microglial barrier around amyloid plaques [212], and thereby reduce plaque load [214, 215]. Other strategies of neutralizing CX3CR1 include antibodies targeted at the receptor or its ligand, or small molecule antagonists such as AZD8797 [216]. However, therapies targeted at CX3CR1 are not without concerns. The likelihood of exacerbating tau hyper-phosphorylation [217, 218], as well as disrupting bacterial clearance by peripheral immune system with systemic suppression of CX3CR1 signaling [219], suggest the importance of searching for approaches to target CX3CR1 signaling specifically in plaque-associated microglia and to minimize potential systemic side effects [220]. Intriguingly, recent reports described that LED-based light flicker stimuli at 40 Hz could promote microglial recruitment, thereby reducing brain amyloid levels in AD transgenic mouse models [221–223].

Studies have linked viral infection with an antimicrobial innate immune response [224, 225], and regulation of AD risk genes [226]. In alignment with these studies, a Phase II trial has been initiated to investigate the efficacy of treating mild AD patients who test positive for serum antibodies for herpes simplex virus 1 or 2, with an anti-viral drug valacyclovir (NCT03282916). The ApoE genotype was taken into consideration during this trial design. The outcome measures include ADAS-cog and ADCS-ADL scores, as well as Aβ and tau burden measured by PET and CSF studies. In addition, changes in cortical thinning on structural MRI, olfactory identification deficits, and antiviral antibody titers from baseline to 78 weeks, will be evaluated. This study will directly address whether virus infections may be etiologic or contribute to the pathology of AD.

## Non-mechanism based approaches

### Symptomatic cognitive enhancers

Symptomatic therapies aiming at cognitive enhancement have been focused on modulating cholinergic and glutamatergic function: the cholinesterase inhibitors (ACHEIs) and a N-methyl-D-aspartate (NMDA) receptor antagonist (memantine). ACHEIs decrease the degradation of acetylcholine released from cholinergic neurons, thereby increasing synaptic transmission. Evidence suggests that ACHEIs moderately improve cognitive and global function status of mild to moderate AD patients [227]. However, the efficacy wanes with long-term treatment due to side effects such as weight loss and syncope [227]. Memantine blocks over-excited NMDA receptors to prevent glutamate release, thereby inhibiting neurotoxicity [228]. It also inhibits and reverses tau hyper-phosphorylation with only mild adverse effects [229]. A combination therapy of memantine with ACHEIs (Namzaric) has been approved for treatment of moderate to severe AD patients [230].

Therapies targeted at other neurotransmitter systems are currently under study. Phase II trials of two compounds: the alpha-7 nicotinic acid agonist, encenicline [231, 232], and the serotonin 5-HT6 antagonist, idalopirdine [233] showed positive results on primary outcome measures (NCT01019421). However, a recent review of results from three Phase III trials of idalopiridine (NCT01955161, NCT02006641, and NCT02006654) suggests no benefit on cognitive function with treatment [234]. Clinical trial of histamine H3 receptor antagonists failed to show consistent benefits [235, 236] (GSK239512, NCT01009255; ABT288, NCT01018875). A Phase II clinical trial of monoamine oxidase (MAO) B inhibitor rasagiline in people with mild to moderate AD is currently ongoing to evaluate its effects on brain metabolism [237] (NCT02359552). Two Phase II clinical trials of ladostigil, a combination of a cholinesterase inhibitor and a MAO B inhibitor, failed to achieve beneficial effects on primary endpoint measures [238–241] (NCT01429623; NCT01354691). However, one trial that targeted delaying MCI conversion to AD, showed a trend towards benefits on selective cognitive tests and brain MRI measure (NCT00000173).

Therapies targeted at synaptic function have also been tested including protein kinase C epsilon (PKCξ) activators, Fyn kinase inhibitors and phosphodiesterase (PDE) inhibitors. Agents activating PKCξ like bryostatin have been tested based on results showing that reduced PKCξ activity is correlated with impaired synaptic and cognitive functions in AD animal models [242]. Moreover, Fyn kinase has been linked to synaptic function and AD pathogenesis. Fyn plays an important role in synaptic plasticity by regulating trafficking of the NMDA glutamate receptor subunits NR2A and NR2B [243–246]. Fyn deficiency mice presented blunted long-term potentiation (LTP) and impaired contextual fear memory function [243, 247]. On the other hand, Fyn has been found to mediate Aβ toxicity through interaction between oligomeric Aβ and the metabotropic glutamate receptor mGluR5 at the post-synaptic plasma membrane [248–250]. Fyn also phosphorylates dendritic tau [249–251]. A preclinical study reported that saracatinib (AZD0530), a Src family kinase inhibitor with high potency for Fyn and Src kinase, rescued synaptic dysfunction and spatial memory impairment in AD transgenic mice [248]. A Phase Ib study of AZD0530 in 24 subjects of mild to moderate AD indicated a reasonable safety and tolerance profile, as well as good BBB penetration [252] (NCT01864655). A Phase II study is currently ongoing in patients of mild AD diagnosis confirmed by amyloid imaging to compare two doses of AZD0530 (100 mg and 125 mg daily for 12 months) versus placebo and the influence of ApoE genotype was taken into consideration during trial design (NCT02167256).

The therapeutic implications of PDE inhibitors as cognitive enhancers in AD have been explored. As key enzymes hydrolyzing secondary messenger cyclic adenosine monophosphate (cAMP) and cyclic guanosine monophosphate (cGMP), PDEs play important roles in regulating signaling pathways critical in brain function. The major challenges of developing PDE inhibitors for AD therapy are narrow dose-response ranges and selection of isoform specific inhibitors. Out of 11 family members, inhibitors targeted at PDE3, 4, 5 and 9 have been tested in clinical trials for AD [253, 254]. Several clinical trial studies of a PDE3 inhibitor cilostazol demonstrated beneficial effects on cognitive function in MCI and AD patients [255–257] (NCT02491268). An ongoing Phase IV trial is studying the effects of cilostazol on subcortical white matter hyperintensities in AD subjects (NCT01409564). For inhibitors of PDE4: HT-0712 (NCT02013310), roflumilast (NCT02051335; NCT01433666; ISRCTN96013814), and BPN 14770 (NCT02648672; NCT02840279), and inhibitors of PDE9: BI 409306 (NCT01343706; NCT02337907) and PF-004447943 (NCT00988598), available results are limited to determine their clinical efficacy at this point [253, 254].

### Therapies and interventions for AD prevention

Similar to stroke prevention, AD prevention becomes the second major wave of effort in the field of neurology. In the paper, we divide the effort on AD prevention into secondary and primary prevention interventions. The key concept of secondary prevention is to initiate the mechanism-based interventions with the hope to treat underlying pathophysiology in order to prevent cognitive symptoms from ever developing [258]. Different from secondary prevention trials, primary prevention trials seek to decrease modifiable risks for AD [259] with strategies including lifestyle interventions, co-morbidity treatments, supplemental and multi-domain interventions.

#### Secondary prevention interventions

Since 2011, global collaborative efforts have embarked on secondary prevention trials. The five large trials include: 1) the API Autosomal-Dominant AD (ADAD), 2) API APOE4 Trial, 3) the DIAN-Trials Unit (DIAN-TU), 4) the Anti-Amyloid Treatment in Asymptomatic Alzheimer’s Disease (A4) trial, and 5) the TOMMORROW Trial. API, DIAN, and A4 have already formed an umbrella group called the Collaboration for Alzheimer’s Prevention (CAP) to maintain regular dialogue about study design and outcome validation. Four of the five trials above have agreed to continue testing the amyloid hypothesis and are actively working together to make sure that the cognitive outcomes are comparable and meaningful [260]. The questions to be addressed include guidelines for safe monitoring of subjects for ARIA [261], timing of interventions for maximal benefit of prevention [260], and innovative cognitive outcomes for approval of investigational drugs for early AD with post-approval monitoring [262].

#### Primary prevention interventions

Primary prevention targeted at specific lifestyle interventions often includes management of cardiovascular disease or metabolic risk factors, changes in diet and exercise, cognitive stimulation or training, and social engagement [260].

Hypertension and hyperlipidemia were two major cardiovascular risk factors targeted in AD and dementia prevention. The Systolic Hypertension in Europe (Syst-Eur) trial, also called vascular dementia project, demonstrated a significant benefit for stroke with reduction of systolic blood pressure by at least 20 mmHg to a goal of below 150 mmHg [263]. The intervention was found to reduce the incidence of dementia by 50%, with AD dementia included as a subcategory. It was calculated that out of 1000 persons treated for hypertension for 5 years, 19 cases of dementia could be prevented. A recent study of participants in the Framingham Heart Study indicates that the incidence of dementia has declined over the course of three decades, which paralleled with observed improvement in cardiovascular health over time [264]. On the other hand, hyperlipidemia as a potential risk factor for AD remains controversial. Several trials have carried out to evaluate the effects of statins in AD. Two trials considered to meet inclusion criteria by the Cochrane review were the Heart Protection Study (HPS) trial and the Prospective Study of Pravastatin in the Elderly at Risk (PROSPER) trial [265] (NCT00939822). However, neither of these two studies demonstrated any beneficial effects of statins on AD prevention or cognitive decline [266, 267].

Diabetes has been another focus of AD prevention. The most prominent trial was the multi-site randomized study called the Action to Control Cardiovascular Risk in Diabetes Trial with Memory in Diabetes (ACCORD-MIND) sub-study, which showed that the group underwent intensive glycemic control of hemoglobin A1c less than 6%, had greater total brain volume measured by MRI, but no differences in the cognitive scores compared to the group of standard of care with hemoglobin A1c at the range between 7 and 7.9% [268] (NCT00182910).

On the other hand, the observation of insulin resistance in AD forms the foundation of evaluating effects of insulin and insulin-sensitizing agents [269, 270]. Early pilot studies of insulin treatment in MCI and AD subjects showed beneficial effects on cognitive function [271–273] (NCT00438568). An ongoing Phase II/III clinical trial in amnestic MCI and mild AD subjects (SNIFF: Study of Nasal Insulin to Fight Forgetfulness) will determine the effects of intranasal insulin on cognitive decline, brain volume loss, and changes in CSF biomarkers (NCT01767909). Insulin-sensitizing agents such as metformin [274] and peroxisome proliferator-activated receptor gamma (PPARγ) agonists like pioglitazone [275], have been advanced into clinical trials of AD (NCT02432287; NCT00982202). Future follow up evaluations of clinical efficacy are needed.

Non-pharmacological interventions like diet, exercise, cognitive training, and vitamin supplement have also been studied in AD prevention trials. The most promising diet intervention has been the Mediterranean diet, rich in fruits and vegetables, combined with olive oil and fish. The Three-City (3C) Study suggests that participants who adhered to the Mediterranean diet had a slower rate of decline on the mini-mental status examination (MMSE), but not the other cognitive tests [276]. Studies have also shown that the Mediterranean-DASH Intervention for Neurodegenerative Delay (MIND) diet may reduce the risk of Alzheimer’s by up to 50%, and the protective effects persist till later time points even when diet recommendations were not followed rigorously [277, 278]. An ongoing preventive trial targeted at 65 years or older overweight individuals without cognitive impairment but with suboptimal diet habits will determine the effects of the MIND diet on cognitive decline and brain neurodegeneration over a 3-year period (NCT02817074).

There have been many studies investigating the association between physical activity and AD. An inverse association has been implicated despite moderate quality of evidence based on literature review of prospective observational and intervention studies [279]. However, current evidence is insufficient to provide detailed recommendations regarding specific physical exercise linked to AD prevention, e.g. type, frequency, intensity and duration of exercise [279]. Overall, it is indicated that physical activities combined with social and cognitive stimulation or diet modification may be more beneficial at reducing risks of AD [279]. For example, in a recent randomized controlled trial known as Fitness for the Aging Brain Study (FABS), subjects with memory complaints but no dementia, who underwent a 6-month exercise program had modest cognitive improvement at 18 months (0.26 points increase on the ADAS-cog score), whereas the control group declined 1.04 points [280] (ACTRN12605000136606). When combined with the Mediterranean diet, physical activity was associated with a significant reduction of AD incidence. For those with a high score for both healthy diet and physical activity, the hazard ratio for AD was 0.65 [281]. Besides physical activity, the effects of cognitive training have also been evaluated. The most robust study to date is the Advanced Cognitive Training for Independent and Vital Elderly (ACTIVE) trial, which provided the strongest evidence for beneficial effects of cognitive intervention in the prevention of cognitive impairment [282] (NCT00298558).

In the past decade or so, more attention has been brought to the use of supplements such as vitamin E, Gingko biloba and omega-3 fatty acids for AD prevention. Based on a recent systematic review, there is no evidence suggesting any beneficial effects of vitamin E on preventing MCI conversion into AD, or improving cognitive function of MCI or AD patient [283]. However, results from a single study of combined vitamin E and memantine therapy in AD subjects suggest that vitamin E may delay functional decline in these patients by 19% per year over the 4-year time period [284, 285] (NCT00235716).

The clinical studies of *Ginkgo biloba* extract, also known as EGb 761, showed no beneficial effects in AD prevention. The Ginkgo Evaluation of Memory (GEM) study of 3069 cognitively normal subjects aged 75 or older and 482 subjects with MCI showed that *Ginkgo biloba* did not prevent cognitive decline in normal aging or MCI subjects during 6 years of follow up [286, 287] (NCT00010803). Another trial called GuidAge had the same results in a group of 2854 subjects with subjective memory complaints who were given the same *Ginkgo biloba* extract for 5 years [288] (NCT00276510).

Omega-3 fatty acids have a complex association with AD, as noted in a recent systematic review, which found that seven out of the 11 observational studies had positive findings, but none of the four clinical trials had any benefit for prevention or treatment of dementia [289]. An example of a clinical trial that has shown the benefits of omega-3 fatty acids is the Memory Improvement with Docasahexaenoic Acid Study (MIDAS) [290] (NCT00278135). Future studies are needed to determine the effects of omega-3 fatty acids in larger clinical trials, and possible multimodal interventions for AD prevention.

Finally, multidomain interventions targeting vascular and lifestyle risk factors as previously described have been tested in AD prevention trials: 1) the Prevention of Dementia by Intensive Vascular Care study (PreDIVA), 2) the Finnish Geriatric Intervention Study to Prevent Cognitive Impairment and Disability (FINGER; NCT01041989) [291], 3) the Multidomain Alzheimer Preventive Trial (MAPT), and 4) the Multimodal Preventive trial for AD (MIND-ADMINI; NCT03249688). These ongoing multi-centered trials emphasize the importance of international collaboration and standardization of study design. They are unique in AD clinical research because these are the first randomized, controlled trials looking into a combination of treatments.

## Future directions for AD drug development

As research in AD progresses, knowledge of underlying AD pathogenesis will guide future drug development effort. In the past two decades or so, we have learned that amyloid may not be the only critical step or the only mechanism of action to be targeted in AD. Tau-modifying agents have been developed, as well as many other agents discussed in this article as summarized in Table 2, which open the door for combination therapy like the case in cancer, cardiovascular and infectious disease treatment [260] Table 2.

The importance of utilizing biomarkers to help diagnosis of preclinical AD has been increasingly recognized in the field [292]. AD biomarkers include key proteins that reflect AD pathology such as amyloid and tau, as well as biomarkers of neuronal injury and regional patterns of abnormalities detected by various imaging modalities that provide indirect evidence of disease development and progression [293, 294]. AD clinical trials may incorporate biomarkers, so that in trial design precise timing of interventions can be elucidated, as well as subgroups of patients for intervention be determined and outcome measures be monitored. For example, Tau PET imaging is now emerging on the scene and could help further refine the preclinical stages of AD, as well as serve as an important outcome measure in AD prevention trials. The ultimate goal is to develop an algorithm of utilizing multiple biomarkers to predict the probability of conversion or progression to dementia in at-risk individuals [293].

Recent failures of several AD clinical trials make us realize that when AD progresses to certain point, excessive neurodegeneration becomes irreversible and aberrant neural networks cannot be repaired by simply reducing amyloid burden or oxidative stress. Current effort has been shifted to AD prevention in the early stages of disease [260, 295]. The recent report of beneficial effects on slowing down disease progression by anti-Aβ antibody BAN2401 in prodromal AD subjects suggests the feasibility of initiating disease-modifying treatments as early as possible. Many other preventative measures as discussed in this article, such as neuroprotection, cognitive enhancement and lifestyle modifications, may synergistically contribute to slow the progression of AD, when intervention(s) begins at the early stages of the disease processes [296]. Other major focuses are multi-targeted drug development and drug repositioning in AD [43].

## Conclusions

AD is a complex and multifactorial disease for which the mechanisms remain to be fully elucidated. The lack of success in the Aβ-centric single target approach is compelling evidence that the paradigm of AD drug design needs to be shifted. As new insights into AD pathogenesis and progression are gained, developing or repurposing drugs with the capacity to target different aspects of the disease pathogenesis at once become promising in AD therapies. In addition, the development of novel biomarkers and imaging tools in AD has advanced dramatically in the past decade. However, the application of these tools in clinical trial practice is yet to be fully optimized. Finally, we are entering the era of “big data”. The concept of precision medicine has been introduced into AD field with the goal to utilize patient-centered approaches focusing on early screening for risks and detection of pathophysiology. Using customized multi-targeted and biomarker-guided strategies, we can achieve both effective and safe preventive therapies, based on the disease characteristics of each individual patient.

## Abbreviations

3C Study: The three city study
7,8-DHF: Flavone 7,8-dihydroxyflavone
A4 Trial: Anti-amyloid treatment in asymptomatic alzheimer’s disease trial
ACCORD-MIND: Action to control cardiovascular risk in diabetes trial with memory in diabetes
ACHEI: Cholinesterase inhibitors
ACTIVE: Advanced cognitive training for independent and vital elderly
AD: Alzheimer’s disease
ADAD: API autosomal-dominant AD
ADCOMS: Alzheimer’s disease composite score
ADCS-ADL: Alzheimer’s disease cooperative study – activities of daily living
APECS: Amyloid-b production and effects on cognition study
APH-1: Anterior pharynx defective 1
ApoE: Apolipoprotein E
APP: Amyloid Precursor Protein (APP)
ARIA: Amyloid-related imaging abnormalities
ARIA-E: Amyloid-related imaging abnormalities edema
Aβ: Amyloid-beta
BACE-1: b-site APP cleaving enzyme 1
BBB: Blood brain barrier
CAP: Collaboration for Alzheimer’s prevention
CBS: Corticobasal syndrome
CDK5: Cyclin dependent kinase 5
CDR-SB: Clinical dementia rating sum of boxes
CNS: Central nervous system
CSF: Cerebrospinal fluid
DIAN-TU: DIAN trials unit
FABS: Fitness for the aging brain study
FAD: Familial Alzheimer’s disease
FDG-PET: Flurodeoxyglucose positron emission tomography
FINGER: Finnish geriatric intervention study to prevent cognitive impairment and disability
GEM: Ginkgo evaluation of memory
GI: Gastrointestinal
HPS: Heart protection study
LTD: Long term depression
LTP: Long term potentiation
MAO: Monoamine Oxidase
MAPT: Multidomain alzheimer preventive trial
MCI: Mild cognitive impairment
MIDAS: Memory Improvement with Docasahexaenoic Acid Study
MIND Diet: Mediterranean-DASH intervention for neurodegenerative delay diet
MIND-ADMINI: Multimodal preventative trial for AD
MMSE: Mini mental state examination
MT: Microtubule
NCT: Nicastrin
NFT: Neurofibrillary tangles
NGF: Nerve growth factor
NIC5–-15: Pinitol
NMDA: N-methyl-D-aspartate
NSAIDs: Non-steroidal anti-inflammatory drugs
NT: Neurotrophins
PEN-2: Presenilin enhancer 2
PHF: Paired helical filaments
PK: Protein kinase
PP: Protein phosphatase
PPARγ: Proliferator-activated receptor gamma
PreDIVA: Prevention of dementia by intensive vascular care study
PROSPER: Prospective study of pravastatin in the elderly at risk
PS: Presenilin
PSP: Progressive supranuclear palsy
RXR: Retinoid X receptor
SNIFF: Study of nasal insulin to fight forgetfulness
Syst-Eur Trial: Systolic hypertension in europe trial
Tg: Transgenic
Trk: Tropomyosin receptor kinase
